# Nerve invasion as an independent predictor of poor prognosis in gastric cancer after curative resection

**DOI:** 10.1097/MD.0000000000030084

**Published:** 2022-08-19

**Authors:** Chunsheng Li, Mingchuan Wang, Xianbin Cheng, Yang Jiang, Huijie Xiao

**Affiliations:** Department of Gastrointestinal Colorectal and Anal Surgery, China-Japan Union Hospital of Jilin University, No. 126, Xiantai Avenue, Changchun, China.

**Keywords:** gastric cancer, perineural invasion, PNI, prognosis

## Abstract

The study aims to reveal the clinical significance of perineural invasion (PNI) for gastric cancer prognosis and determine the risk factors of PNI in gastric cancer. This study retrospectively analyzed 350 patients who were diagnosed with GC and underwent curative surgical resection. Variables used to analyze survival included gender, age, degree of differentiation, T classification, lymph node metastasis, lymphovascular invasion, nerve invasion, mucinous adenocarcinoma component, and signet ring cell carcinoma component. The tumors of all patients were surgically resected. All resected specimens were stained with hematoxylin-eosin and immunohistochemical. The data for the patient’s lymphovascular invasion and PNI came from the collected pathological reports. The results of the survival analysis showed that T staging (*P* < .001), lymph node metastasis (*P* < .001), lymphovascular invasion (*P* = .013), PNI (*P* = .001), and signet ring cell carcinoma components (*P* = .046) affect the survival time and have a statistically significant difference. Multivariate analysis indicated that the positivity of PNI was an independent prognostic factor (*P* = .014). T staging (*P* = .006) and lymph node metastasis (*P* = .013) were independent prognostic parameters too. Using the Spearman correlation analysis, the following clinicopathological indicators were associated with PNI positivity, such as tumor differentiation, T staging, lymph node metastasis, vascular invasion, and signet ring cell carcinoma components (*P* < .05). PNI is an independent marker of poor prognosis in patients with gastric cancer.

## 1. Introduction

Gastric cancer (GC), referring to a malignant tumor derived from the epithelium of the gastric mucosa, is one of the most common malignant tumors of the digestive tract. In recent decades, ignoring that the incidence of gastric cancer has steadily declined globally, gastric cancer is still the fifth most widespread cancer, with more than 1 million cases in 2018, of which nearly two-thirds occurred in developing countries.^[1]^

The statistical results of many studies show that many factors affect the prognosis of gastric cancer, including tumor differentiation type, infiltration depth, number of lymph node metastasis, etc. With the continuous improvement and development of anatomy, molecular biology, and postoperative pathological examination, perineural invasion (PNI) in gastric cancer has gradually entered the keen vision of scholars. PNI refers to the charge of peripheral nerve fibers by gastric tumor cells. It is a distinctive feature of various solid tumors (including pancreatic cancer, colorectal cancer, and prostate cancer), indicating a poor prognosis.^[2–4]^The statistical results showed that the total proportion of postoperative pathological nerve infiltration in gastric cancer patients was 40.5%. Some studies have shown^[5–8]^ that the occurrence of nerve infiltration may be related to many factors, such as the depth of local tumor invasion, lymph node metastasis, vascular and lymphatic invasion, TNM staging, etc. As the depth of tumor invasion increases, the incidence of PNI also gradually increases.

Along with the occurrence of nerve invasion, the invasion of blood vessels and lymphatic vessels gradually increases. The survival rate of malignant tumors is positively correlated with local tumor invasion, distant metastasis, and TNM staging. Therefore, we believe that the occurrence of PNI in gastric cancer may be related to prognosis and may serve as a breakthrough in assessing the prognosis of gastric cancer. In recent years, PNI as a new biological characteristic has also attracted more and more attention among researchers. However, only a few studies have looked into how perineural invasion affects the prognosis of gastric cancer.

Furthermore, previous data on the effect of PNI on the prognosis of gastric cancer patients is contradictory, and little is known about PNI risk factors in gastric cancer patients.To overcome the limitations of previous studies on the relationship between PNI and the prognosis of gastric cancer, which come from the small patient cohort and short follow-up period, we retrospectively analyzed 350 patients diagnosed with GC who underwent radical resection. The study aims to reveal the clinical significance of PNI for the prognosis of gastric cancer and determine the risk factors for PNI in gastric cancer. This is critical for guiding the clinical formulation of a reasonably comprehensive treatment plan, improving treatment efficacy, and improving gastric cancer prognosis.

## 2. Methods

### 2.1. Patient population

This study retrospectively analyzed 350 patients diagnosed with GC who underwent curative surgical resection at China-Japan Union Hospital of Jilin University from March 31, 2015, through March 31, 2019. Among them, 37 cases were lost to follow-up, and the follow-up rate was 89.4%. The remaining 313 patients obtained survival status through telephone inquiries, outpatient and inpatient reviews, and were included in the survival analysis. Survival time is defined as the time after surgery to any cause of death, and the final follow-up deadline is March 31, 2019. Among the 313 patients with gastric cancer, 67.1% were males and 37.9% were females. They were between 28 and 85 years old, with an average age of 60 years. The median follow-up time was 52 months (0.5–60 months). Variables used to analyze survival included gender, age, degree of differentiation, T classification, lymph node metastasis, lymphovascular invasion, nerve invasion, mucinous adenocarcinoma component, and signet ring cell carcinoma component. All surgically resected specimens were stained with hematoxylin-eosin and immunohistochemical staining. The data for the patient’s lymphovascular invasion and PNI came from the collected pathological reports. PNI is defined as a tumor close to the nerve and involving at least 33% of its circumference or tumor cells within any of the 3 layers of the nerve sheath. After completing data collection, we analyzed the relationship between postoperative pathological indicators and the survival rate of gastric cancer patients. Then, we looked at the relationship between PNI and a lot of different things about the people who had the cancer. This included things like age, gender, the level of differentiation, the T-cell type of cancer, lymph node metastasis, lymphovascular invasion, nerve invasion, mucinous adenocarcinoma, and signet ring cell carcinoma.

### 2.2. Inclusion and exclusion criteria

Inclusion criteria: (1) patient with primary gastric cancer was treated with surgery in our hospital, and the pathological diagnosis was confirmed as gastric adenocarcinoma after surgery; (2) no radiotherapy, chemotherapy, surgery, interventional therapy, Chinese medicine, or other treatments were performed prior to diagnosis; and (3) patient clinical data can be entirely collected as needed.

Exclusion criteria: (1) remote metastasis or invasion of nearby organs, or palliative surgery if the tumor cannot be removed entirely; (2) combined with other tumors or a history of malignant tumors, such as liver cancer, gastric stump cancer, etc; (3) Pathological diagnosis of squamous cell carcinoma, adenosquamous carcinoma, signet ring cell carcinoma, small cell carcinoma, lymphoma, stromal tumor, or patients with an unknown tissue type.

### 2.3. Statistical analysis

SPSS 24.0 software was used for data entry and statistical analysis. Patient characteristics were compared using t-tests for continuous variables and Chi-squared or Fisher exact tests for categorical variables. The Kaplan–Meier method was used to evaluate 5-year overall survival (OS) and plot broad survival curves; the log-rank test assessed the difference in survival curves. The Cox proportional hazards regression model analyzed the predictors with *P* < .05 in the log-rank test. OS was defined as the period from the time of surgery to the date of death or last follow-up. To select final indicators of PNI, all candidate parameters with a *P* < .05 in Spearman correlation analysis were included in a multivariate logistic regression model. All *P* values were 2-sided and a value of < 0.05 indicated statistical significance.

## 3. Results

A total of 350 patients who had undergone radical gastrectomy for gastric cancer were included in this study, of which 313 patients completed a 5-year follow-up; 115 patients were women and 235 were men. The median age was 62 years, ranging from 28 to 85 years. 134 patients were younger than 60 years (38.3%). Based on T staging, 109 (31.1%) patients were classified as T1T2, and 241 (68.9%) as T3T4. Of the 350 patients, 171 (50.1%) and 172 (49.9%) surgical specimens were classified as PNI positive (PNI (+)) and PNI negative (PNI (-)), respectively. The clinical and pathologic characteristics of the patients are shown in Table 1.

**Table 1:** Clinicopathologic characteristics of the patients.

Clinical feature	n	%
Gender		
Male	235	67.1
Female	115	32.9
Age (yr)		
<60	134	38.3
≥60	216	61.7
Tumor grade		
Moderately + well	66	18.9
Poorly	284	81.1
T stage		
<3	109	31.1
≥3	241	68.9
N stage		
<1	120	34.3
≥1	230	65.7
Perineural invasion		
No	172	50.1
Yes	171	49.9
Lymphovascular invasion		
No	138	40.2
Yes	205	59.8
Mucinous cancer		
Yes	308	88.0
No	42	12.0
Signet ring cell carcinoma		
No	254	72.6
Yes	96	27.4

**Figure 1.**
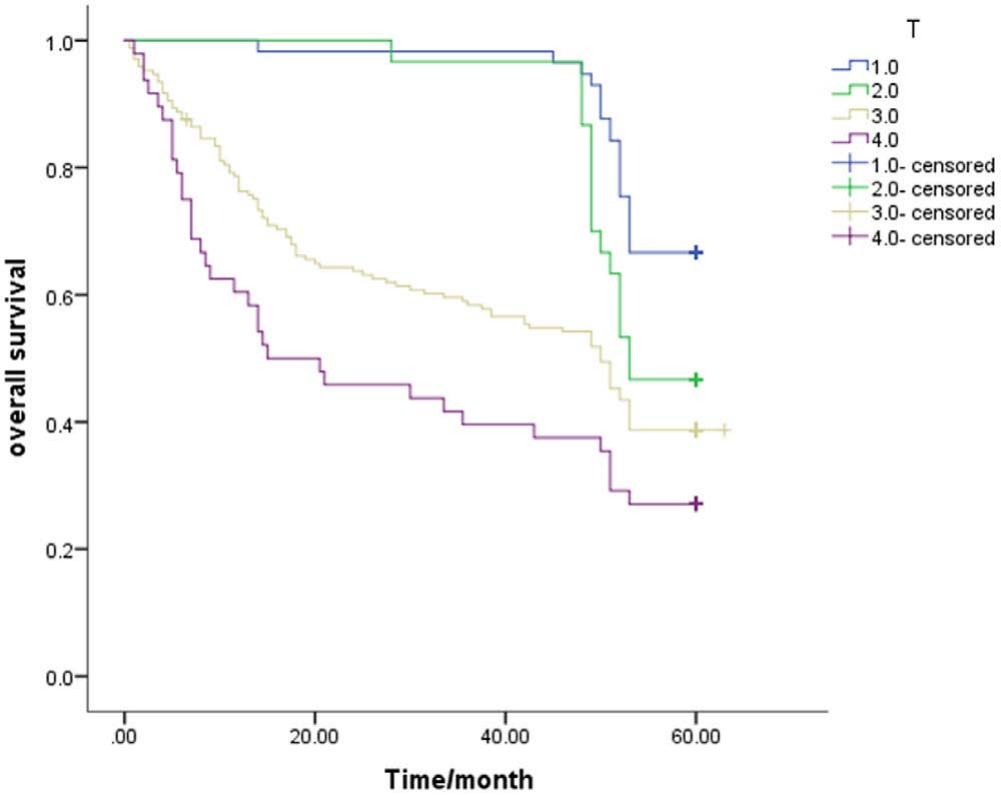
Unadjusted Kaplan-Meier survival analysis for T stage related to overall survival in all patients.

**Figure 2.**
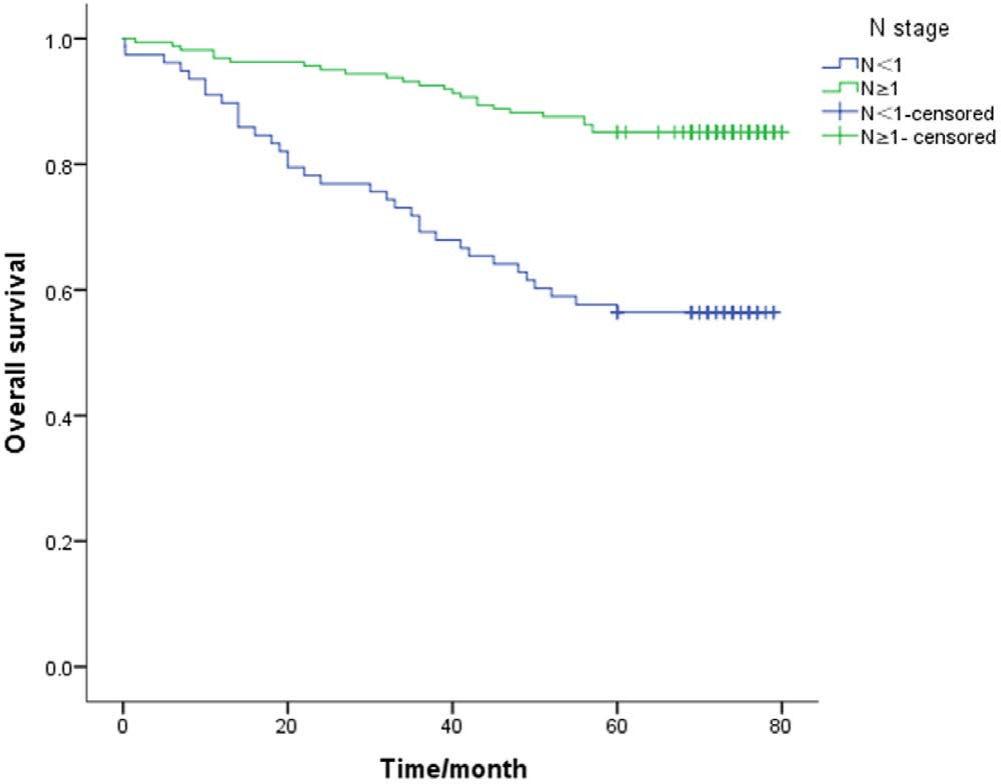
Unadjusted Kaplan-Meier survival analysis for N stage related to overall survival in all patients.

**Figure 3.**
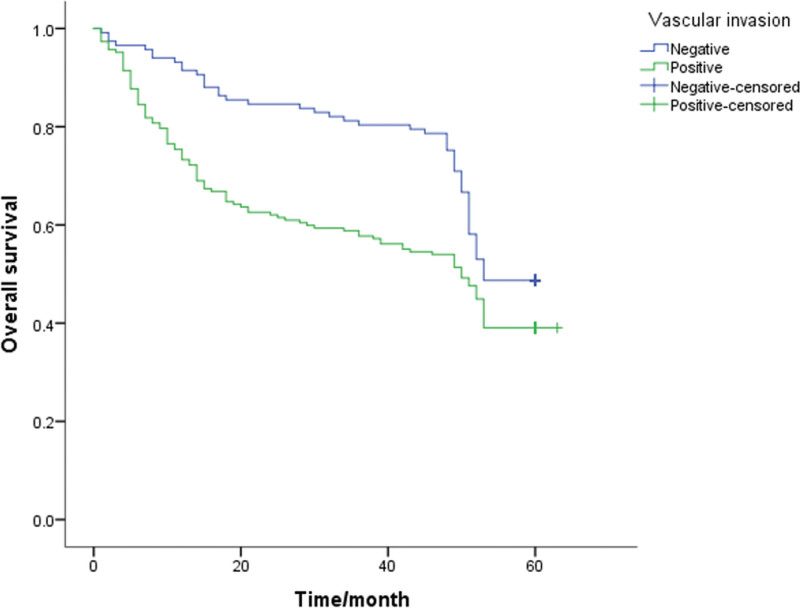
Unadjusted Kaplan-Meier survival analysis for vascular invasion related to overall survival in all patients.

**Figure 4.**
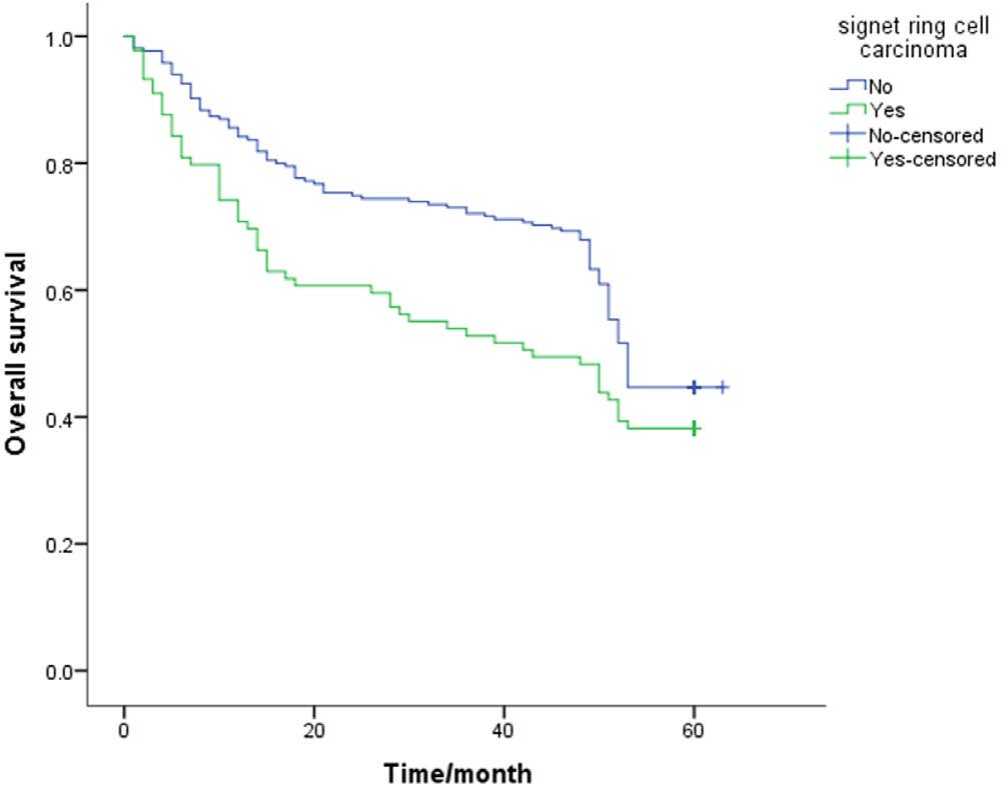
Unadjusted Kaplan-Meier survival analysis for signet ring cell carcinoma related to overall survival in all patients.

**Figure 5.**
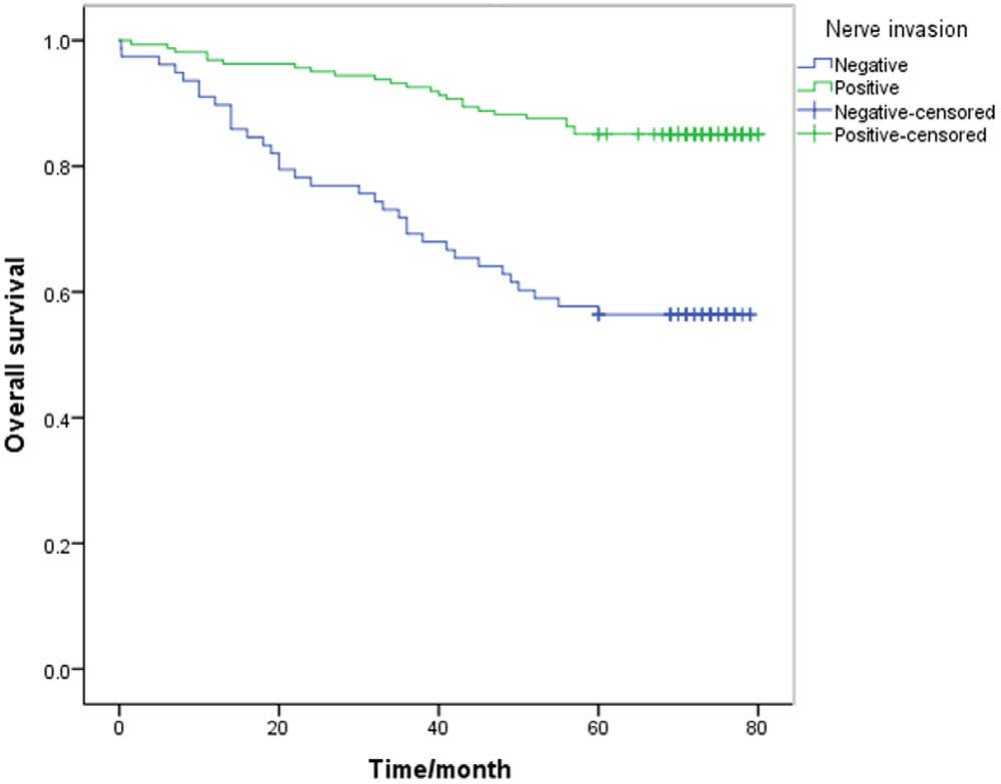
Unadjusted Kaplan-Meier survival analysis for nerve invasion related to overall survival in all patients.

A Kaplan-Meier survival analysis was performed on each clinicopathological parameter. The results of the survival analysis showed that T staging (*P* < .001), lymph node metastasis (*P* < .001), lymphovascular invasion (*P* = .013), PNI (*P* < .001) and signet ring cell carcinoma components (*P* = .046) affect the survival time and have a statistically significant difference (Table 2). All the above factors are risk factors that affect the prognosis of patients. The survival analysis results also showed that the differences in survival time caused by gender, age, tumor differentiation, and mucinous adenocarcinoma components were not statistically significant (*P* > .05, Table 2). The 5-year survival rate of patients with PNI (+) was 32.1%, and that of patients without PNI was 54.4%. The 5-year survival rates of T1-2 and T3-4 patients were significantly different (*P* < .05; 61.7% and 36.1%, respectively). Also, the survival rates of patients with negative and positive lymph node metastasis were 60.8% and 35.5%, respectively. The survival curve is shown in Figure 1–5.

**Table 2:** Univariate analyses of factors for 5-year overall survival (OS).

	Over survival		Log-Rank	P
		95%CI		
Gender				
Male	41.495	38.549–44.441	1.252	0.263
Female	43.367	38.680–48.054		
Age				
<65	42.890	39.884–45.896	0.847	0.357
≥65	40.708	36.298–45.118		
Mucinous cancer				
Yes	40.778	40.292–45.872	2.676	0.102
No	43.082	40.292	45.872	
Tumor grade				
Moderately + well	48.877	44.743–53.012	0.632	0.427
Poorly	41.239	38.264–44.213		
T stage				
1	56.368	54.423–58.214	31.124	<0.001
2	54.000	51.510–56.490		
3	39.296	35.677–42.896		
4	29.073	22.357–35.788		
N stage				
<1	53.854	51.555–56.154	23.160	<0.001
≥1	37.034	33.734–40.33		
Signet ring cell carcinoma				
No	45.442	42.553–48.331	3.979	0.046
Yes	35.640	30.714–40.567		
LVI				
Positive	38.679	35.168–42.190	6.230	0.013
Negative	48.205	45.063–51.347		
Nerve invasion				
Positive	33.648	30.014–37.281	27.814	<0.001
Negative	52.007	49.232–54.782		

We performed multivariate analysis with the Cox regression method to further evaluate the prognostic significance of PNI and the other clinicopathological factors, which showed significant differences in the log-rank test. Multivariate analysis indicated that the positivity of PNI was an independent prognostic factor (*P* = .014, OR = 0.642, 95%CI 0.451–0.916). What’s more, T staging (*P* = .006) and lymph node metastasis (*P* = .013) were independent prognostic parameters, too. Unexpectedly, the data in this article show that lymphovascular invasion (*P* = .066) and signet ring cell carcinoma component (*P* = .199) are confounding factors, not independent risk factors, that affect the prognosis of gastric cancer patients. The results are shown in Table 3.

**Table 3:** Multivariate analyses of factors for 5-year overall survival (OS).

	OR	95%CI	*P*
T stage	1.347	1.089–1.667	0.006
N stage	0.555	0.349–0.883	0.013
Signet ring cell carcinoma	0.808	0.583–1.119	0.199
LVI	1.438	0.976–2.118	0.066
Nerve invasion	0.642	0.451–0.916	0.014

Using the Spearman correlation analysis, the following clinicopathological indicators were found to be associated with PNI positivity, such as tumor differentiation, T staging, lymph node metastasis, vascular invasion, and signet ring cell carcinoma components (*R* = 0.298, 0.518, 0.454, 0.414, 0.166, respectively; *P* < .05). On the other hand, the relationship between PNI positivity and sex, age, and mucinous adenocarcinoma composition was not detected. The Chi-square test showed that poor differentiation, T3-4 stage, positive lymph node metastasis, signet ring cell carcinoma, and positive lymphovascular invasion were all risk factors for PNI in gastric cancer (X² = 30.449, 92.104, 70.680, 9.422, 58.729, respectively; *P* < .05). The differences between the parameters that were found to be significant in the Spearman correlation analysis were then evaluated by logistic regression analysis. Logistic regression analysis showed that poor differentiation, T3-4 stage, lymph node metastasis and lymphovascular invasion were independent risk factors for PNI in gastric cancer (OR = 2.882, 8.392, 2.695, 2.186, respectively; *P* < .05). In Table 4, the associations between PNI and clinicopathological factors are shown.

**Table 4:** The associations between PNI and clinicopathological factors.

		Perineural invasion			
Clinical feature	n	Positive	Negative	χ²	P
Gender					
Male	232	120	112	1.003	0.317
Female	111	51	60		
Age (yr)					
<60	131	74	57	3.732	0.053
≥60	212	97	115		
Tumor grade					
Moderately + well	64	12	52	30.449	0.000
Poorly	279	159	120		
T stage					
<3	104	11	93	92.104	0.000
≥3	239	160	79		
N stage					
<1	116	21	95	70.680	0.000
≥1	227	150	77		
Mucinous cancer					
Yes	301	147	154	1.017	0.313
No	42	24	18		
Signet ring cell carcinoma					
No	250	112	138	9.422	0.002
Yes	93	59	34		
Lymphovascular invasion					
No	138	34	104	58.729	0.000
Yes	205	137	68		

## 4. Discussion

In recent years, with the continuous progress of postoperative pathology, perineural invasion (PNI) has entered people’s field of vision and relevant studies have emerged one after another. The mechanism, definition, classification and clinical significance of perineural invasion of malignant tumors have been continuously recognized.

PNI can occur in various cancers, and its definition evolved from the original “tumor growth along a nerve.” Panizza BJ et al divides PNI into 2 categories: pathologists incidentally find in patients with no neurological symptoms and those that can be detected on magnetic resonance imaging (MRI) scans and patients with clinical symptoms such as sensory impairment and paralysis. The former occurs when the tumor enters peripheral nerve endings located in or near cancer. The latter occurs when the cancer spreads along nerves far from cancer, using the lamellar sheath as a barrier, known as “clinical PNI” or “preserve diffusion (PNS),”^[9]^ Basakis (1985)^[10]^ first described PNI in SCC as “invading, circumventing and passing through” nerves. Currently, PNI is defined as “a tumor that is closely adjacent to the nerve and must involve at least 33% of the peripheral nerve, or the tumor cells are located in any of the 3 layers of the nerve sheath”.^[11]^ This definition includes both peri-nerve and intraneural invasions; even among pathologists, there is disagreement about the interpretation of PNI in tissue specimens.^[12]^

The pathogenesis of PNI, especially the exact pathological mechanism of PNI in different types of cancer, is not very clear. A century has passed since PNI was first proposed, and scientists’ understanding of its pathogenesis is constantly changing.^[13]^ The traditional theory of the pathogenesis of PNI is that tumor cells passively spread along the connective tissue covering the nerve or through the perforated blood vessels of the nerve bundle, where resistance is least.^[10]^ Some scholars attribute the pathogenesis to the close anatomical relationship between the tumor and the nerve plexus.^[14]^ For example, Murakawa et al^[15]^ and Nagakawa et al^[16]^ believed that the high positive rate of PNI in pancreatic and biliary tract tumors might be due to the close anatomical structure of pancreatic, biliary tract nerves and celiac nerve plexus. However, the incidence of PNI was not high in rectal cancer patients, despite the proximity of tumors to the presacral autonomic plexus. Kameda et al^[17]^ believed fewer fascicular layers in the terminal nerve but more in the central nerve. Therefore, tumor cells are prone to invade the lamellar sheath via terminal nerves. However, recent studies have pointed to the positive role of the tumor microenvironment in PNI and demonstrated that PNI is caused by the interaction of mutual signals between cancer cells, stromal cells and nerve cells.^[11,18]^ Cancer cells have an innate ability to migrate actively along nerves, which is known as neural tracing. This mechanism is supported by a variety of molecules, including nerve growth factors (NGF) secreted by tumor cells and other nontumor cells in the tumor microenvironment, glial cell lineage-derived neurotrophic factors (GDNF), nerve cell adhesion molecules (NCAM), matrix metalloproteinases (MMPs) and chemokines.^[19]^ The role of chemokines and their receptors in malignant tumors has recently received significant attention among these molecules. According to previous reports, the prevalence of PNI disease in patients with gastric cancer ranged from 31.7% to 65.0%. In this group of cases, the prevalence of PNI in gastric cancer was 49.9%, which further supported the results of previous studies. Previous studies have shown that the detection rate of PNI can be significantly increased by immunohistochemical staining with S-100 or laminin.^[20,21]^ Therefore, we speculated that the actual incidence of PNI in gastric cancer patients without distant metastasis would be higher in the case of a low detection rate of early gastric cancer. PNI positive cases in surgically resected gastric cancer specimens may also become more common because of better detection technology and more experience by pathologists, as well.

The 5-year survival rates of PNI positive and PNI negative patients were 32.1% and 54.4%, respectively. At the end of the follow-up period, more than half of the PNI negative patients had survived (the median survival time was not provided).Our results were consistent with those of Aurello et al and Bilici et al, as well as Tanaka et al, that PNI positive patients had worse survival outcomes than those without PNI.^[7,22,23]^ Duraker et al suggested that although the positive rate of PNI was 59.6% and the incidence of PNI increased with the progression of gastric cancer, PNI did not provide any additional prognostic information compared to classical parameters because PNI did not have independent prognostic significance in multivariate Cox proportional risk model analysis.^[6]^ However, using Kaplan-Mier survival analysis, data in this paper showed that there was a statistical difference between positive and negative PNI in survival (*P* < .01, χ²=28.219), that is, PNI was a risk factor affecting the survival of gastric cancer patients. Furthermore, multivariate Cox proportional risk model analysis showed that neurologic invasion was an independent risk factor for postoperative survival (HR = 1.652, *P* = .006).

Spearman correlation analysis showed that lymphovascular invasion was correlated with nerve invasion (*R* = 0.414, *P* < .05) and the χ² test showed that positive lymphovascular invasion was a risk factor for PNI in gastric cancer (X² = 58.729, *P* < .05). Further analysis, performed by Logistic regression analysis, showed that positive lymphovascular invasion was an independent risk factor for PNI in gastric cancer (OR = 2.186). Previous studies have reported a strong association between lymphovascular invasion infiltration and PNI.^[23–25]^ The abundance of lymphatic networks around the nerve and the direct infiltration of lymph vessels and nerves by cancer cells may partially explain why PNI and lymphovascular invasion are detected in excised specimens. PNI positive is significantly associated with lymphovascular invasion, and we speculate that PNI is not only the result of direct infiltration, but also may occur through invasion of lymphatic vessels and veins around the nerve. Seki et al^[13]^ also pointed out that PNI in cholangiocarcinoma was significantly correlated with lymphatic and venous invasion and proved that PNI in cancer cells was not only the result of direct invasion, which was consistent with our results and speculation. In addition, we investigated the association between PNI and other clinicopathological features, and results showed that PNI was significantly associated with a range of adverse clinicopathological factors, including tumor differentiation, T stage, lymph node metastasis, and signet ring cell carcinoma components. Therefore, patients who are PNI positive are more likely to have more aggressive tumor features than those who are PNI free.

Regrettably, in this work, there appear to be some limitations. In this analysis, we did not evaluate the association between peritoneal or hepatic metastasis and PNI positivity because patients with peritoneal and distant metastasis at the time of diagnosis were excluded from the study, and only patients without distant metastasis were included. Therefore, our study is noteworthy for determining the prognostic significance of PNI in patients with radical gastrectomy without metastasis, and prospective studies should confirm our results.

In conclusion, PNI is an independent marker of poor prognosis in patients with gastric cancer. The discovery of this sensitive marker in patients with gastric cancer who have undergone radical gastrectomy and are at high risk of recurrence will provide helpful information for postoperative follow-up planning and intensive adjuvant chemotherapy. We predict that this is related to the sample size; the difference will become significant as the sample size increases.

## Author contributions

HJX conceived, designed and did statistical analysis & editing of manuscript; CSL and MCW did data collection and manuscript writing; XBC and YJ did review and final approval of manuscript.
